# Early detection and late cognitive control of emotional distraction by the prefrontal cortex

**DOI:** 10.1038/srep10046

**Published:** 2015-06-12

**Authors:** Javier García-Pacios, Pilar Garcés, David Del Río, Fernando Maestú

**Affiliations:** 1 Laboratory of Cognitive and Computational Neuroscience. Center for Biomedical Technology (Technical University of Madrid and Complutense University of Madrid). Campus de Montegancedo. 28223, Pozuelo de Alarcón, Madrid, Spain; 2 Department of Psychology. Faculty of Health Sciences. Camilo José Cela University, Madrid. C/ Castillo de Alarcón, 49, Urb. Villafranca del Castillo. 28692, Madrid, Spain; 3 Department of Basic Psychology II. Complutense University of Madrid. Campus Somosaguas. 28223, Pozuelo de Alarcón, Madrid, Spain

## Abstract

Unpleasant emotional distraction can impair the retention of non-emotional information in working memory (WM). Research links the prefrontal cortex with the successful control of such biologically relevant distractors, although the temporal changes in this brain mechanism remain unexplored. We use magnetoencephalography to investigate the temporal dynamics of the cognitive control of both unpleasant and pleasant distraction, in the millisecond (ms) scale. Behavioral results demonstrate that pleasant events do not affect WM maintenance more than neutral ones. Neuroimaging results show that prefrontal cortices are recruited for the rapid detection of emotional distraction, at early latencies of the processing (70-130 ms). Later in the processing (360-450 ms), the dorsolateral, the medial and the orbital sections of the prefrontal cortex mediate the effective control of emotional distraction. In accordance with the behavioral performance, pleasant distractors do not require higher prefrontal activity than neutral ones. These findings extend our knowledge about the brain mechanisms of coping with emotional distraction in WM. In particular, they show for the first time that overriding the attentional capture triggered by emotional distractors, while maintaining task-relevant elements in mind, is based on the early detection of such linked-to-survival information and on its later cognitive control by the prefrontal cortex.

Emotion and cognition interact in the human brain in order to develop a complex and adaptive behavior. According to some theories, emotional stimuli preferentially recruit cognitive resources^1^,^2^,^3^, as they contain information that is closely linked to survival^4^,^5^,^6^. This preferential access to our cognitive system could be interpreted as a mechanism developed to prepare us to effectively process biologically relevant information, so that we are finally able to build up and exert more adaptive responses. In the memory domain, such an effect has been consistently observed^7^,^8^, and emotional memories have been reported as more vivid^9^, accurate^10^ and resilient to time^11^,^12^,^13^ than neutral memories. However, such a preferential access of emotional stimuli might be problematic when we are engaged in a relevant memory process, as our cognitive resources may be depleted in favor of emotional information. Several studies have shown that emotional information, specifically unpleasant emotional stimuli, can impair the retention of task-relevant neutral information in short term memory^14^,^15^,^16^,^17^, and that individual differences in executive functioning as well as in the cognitive control of the emotional aspects of irrelevant information may account for differences in the ability to cope with emotional distraction^16^,^18^,^19^.

Over the last 10 years, a series of fMRI studies have been devoted to disentangling the brain mechanisms that mediate such cognitive control of emotional distraction in WM. Most of these studies identified a dissociable pattern of activity between dorsal cortical regions, including the dorsolateral prefrontal cortex (DLPFC) and the lateral parietal cortex (LPC), and ventral brain areas, including the orbitofrontal cortex (OFC), the ventrolateral prefrontal cortex (VLPFC), the occipitotemporal cortex (OTC) and the amygdala^7^. Specifically, unpleasant emotional distraction seems to produce a decreased activity over dorsal brain areas which are known to be related to executive processes implicated in attentional processes and active maintenance of information in WM^20^,^21^,^22^,^23^,^24^,^25^. This reduction of activity has been interpreted as the cause of the impairment in the maintenance of task-relevant information observed at the behavioral level. Besides, unpleasant emotional distraction enhances activity in ventral cortical and subcortical regions, which has traditionally been related to emotional processing and emotional regulation^26^,^27^,^28^,^29^. Thus, increases in ventral activity due to processing of emotional distraction appear to exert a bottom-up modulation over dorsal brain regions, reallocating processing resources^30^ and finally impairing the behavioral performance. Moreover, this dorsal-ventral dissociation was found to be specific for emotional distraction^18^.

Nevertheless, WM maintenance is not affected by every single emotional distractor, so our cognitive control mechanisms seem to be able to override such negative bottom-up influence. Specific regions over that ventral emotional processing system, such as the VLPFC, which are widely related to emotional regulation processes^29^,^31^,^32^, have been found to be critically involved in coping with emotional distraction in WM^14^,^15^,^16^,^17^,^19^,^33^. Indeed, activation over those ventral prefrontal regions during emotional distraction processing seems to benefit WM maintenance of task-relevant information^14^,^16^,^19^,^33^.

Although all these studies have established the brain areas that underlie the mechanism that allow us to cope with biologically relevant distraction, the temporal dynamics of this process remain unexplored. In the present study, we use MEG to characterize the spatio-temporal patterns of the brain activity that underlie the cognitive control mechanisms involved in coping with emotional distraction. We also include pleasant emotional pictures in our design, as their potential effect as distractors in WM has not been addressed.

Based on previous evidence showing an early processing of emotional stimuli^34^,^35^,^36^,^37^, we predict that both, pleasant and unpleasant stimuli, but especially the latter, would increase the brain response at early latencies of distraction processing, when compared with neutral stimuli. Since such an early activation has been reported in other kinds of tasks, in which the emotional stimuli do not have to be controlled, we also hypothesize that the effective overriding of emotional distractors would occur later in the processing, and that such cognitive control would be mediated by higher activation of prefrontal cortices, especially in the DLPFC and in the VLPFC.

## Results

### Working memory performance

As expected, Friedman’s test revealed a significant main effect of condition in WM accuracy (hits + correct rejections) [χ^2^(3) = 12.21, *p* = .001)]. Wilcoxon’s test for pairwise comparisons revealed that accuracy after unpleasant distraction (77.5%) was lower than after pleasant (80.41%) (*p* = .008) and neutral (79.79%) (*p* = .03) distraction. No differences were found between neutral and pleasant distraction (*p* = .57). These results were confirmed when a repeated measures ANOVA was computed on the rank transformation of the original data^38^ [F(2, 13)=8.55, *p* = .004); pleasant > unpleasant (*p* = .006); neutral > unpleasant (*p* = .03); pleasant = neutral (*p* = .39)]. This pattern of differences in WM performance also appeared when corrected recognition scores (hits rate - false alarms rate) were used as dependent variables [χ^2^(3) = 8.13, *p* = .01); pleasant > un*p*leasant (*p* = .01); neutral > unpleasant (*p* = .004); pleasant = neutral (*p* = .91)], and was also confirmed when a repeated measures ANOVA was computed on the rank transformation of the original corrected recognition scores [F(2, 13) = 6.7, *p* = .01); pleasant > unpleasant (*p* = .02); neutral > unpleasant (*p* = .003); pleasant = neutral (*p* = .89)].

As each one of the 144 interfering pictures (48 pictures per condition) was used two times in each condition (see Procedure), one might be concerned about the possibility that a specific distraction might have produced less interference the second time it appeared than the first one, due to potential habituation effects. Although there was a minimum of thirty trials between the two presentations of the same interfering picture, we compared the mean accuracy for distractors presented for the first and second time, for each condition separately. Wilcoxon’s tests showed that distracting pictures produced the same amount of interference both times they were presented as distractors for pleasant (*p* = .62), neutral (*p* = .28) and unpleasant (*p* = .88) conditions.

### Subjective emotional ratings

As expected, Friedman’s test revealed a significant main effect of affective category in subjective valence ratings [χ^2^(2) = 30.00, *p* < .0000003)], and Wilcoxon’s test for pairwise comparisons showed that pleasant pictures were rated as the most pleasant followed by neutral pictures, and unpleasant pictures rated as the least pleasant [mean valence ratings: 7.14, sd = 0.52 (pleasant), 5.09, sd = 0.52 (neutral), 2.23, sd = 0.82 (unpleasant), *p* = .001 for all comparisons]. Arousal ratings also varied as a function of affective category [χ^2^(2) = 25.20, *p* < .000003)], with pleasant and unpleasant pictures rated as more arousing than neutral pictures [mean arousal ratings: 5.35, sd = 1.20 (pleasant), 2.27, sd = 0.5 (neutral), 6.48, sd = 0.48 (unpleasant), *p* = .001 for both comparisons]. Unpleasant pictures were rated as more arousing than pleasant pictures (*p* = .006) (see Table 1 for mean subjective values).

### Event-related fields

**Figure 1:**
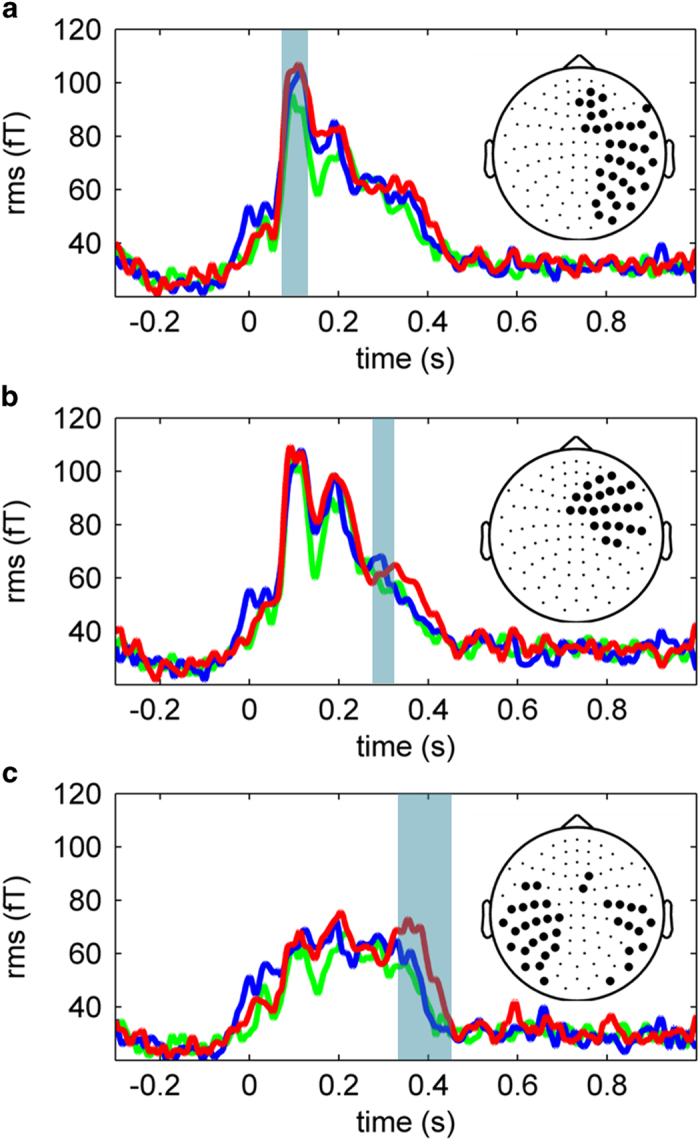
Root-mean-square of grandaverage ERF waveforms in significant clusters of sensors as detected by permutation statistics between 70-130 ms (**a**), 280-320 ms (**b**) and 360-455 ms (**c**). Insets depict sensor cluster locations. Blue, green and red lines represent pleasant, neutral and unpleasant distraction, respectively.

The non-parametric cluster-based analysis performed on sensor-level data revealed three significant clusters of sensors that arose at three different temporal windows, indicating that the neuromagetic response to distracting emotional stimuli varied across conditions and in time. The first significant cluster (*p* = 0.01) involved 35 right sensors and emerged between 70 and 130 ms after the onset of the distracting picture. The second cluster (*p* = 0.03) emerged about 280–320 ms, across 23 right anterior sensors. Finally, a third significant cluster (*p* = 0.03) was composed by 36 sensors bilaterally distributed and arose between 360 and 455 ms. Figure 1 plots the time course of the average neuromagnetic response for each significant cluster (see Fig. 1).

Although the topographical distribution at sensor space does not faithfully represent the actual distribution of the underlying cortical sources, this first analysis showed significant effects of distraction type and pointed out the specific time windows where these differences emerged. Thus, source reconstruction was performed for these time intervals, to investigate the changes in brain activity originating the observed ERF differences.

### Source-space activity

Results from pairwise comparisons in each of the significant time windows identified at sensor level revealed differences between emotional and neutral distraction at early (70–130 ms) and medium (280–320 ms) latencies and between unpleasant and both, pleasant and neutral distraction, at late (360–455 ms) latencies at the source level.

### Early prefrontal enhanced activity by emotional distraction

**Figure 2:**
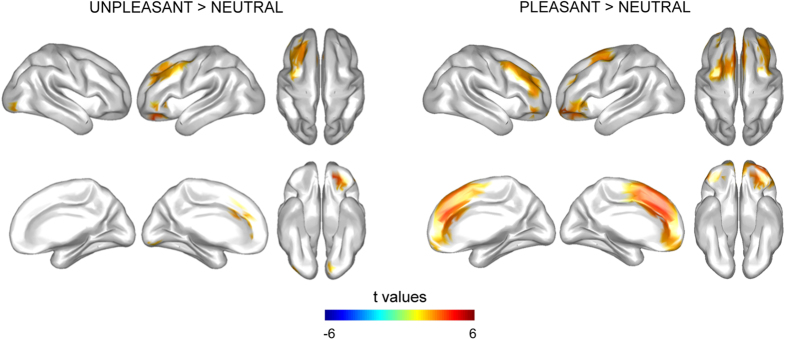
Cortical distribution of statistical differences in brain activity between 70–130 ms. Emotional distractors enhanced brain activity in the DLPFC, the VLPFC, the OFC, the MPFC and the PosFC. Unpleasant distraction also produced increased activity in OC when compared with neutral distraction.

Both emotional distractions produced significantly increased brain activity about 70–130 ms when compared to neutral distraction. Particularly, pleasant distractors enhanced brain activity in a cortical bilateral cluster (*p* = 0.0008) composed by a number of frontal regions, including the DLPFC, the VLPFC, the OFC, the medial prefrontal cortex (MPFC) and the posterior frontal cortex (PosFC). Unpleasant distractors also increased activity in two clusters, one of them over left frontal cortices including the DLPFC, the VLPFC, the OFC, the MPFC and the PosFC (*p* = 0.01), while the other one (*p* = 0.04) was composed of the occipital cortex (OC) (see Fig. 2 for cortical distribution of statistical differences in brain activity and Table S1 for specific cortical regions included in the clusters, as defined in the Automated Anatomical Labeling Atlas (AAL atlas)^39^).

### Increased temporal activation by unpleasant distraction

**Figure 3:**
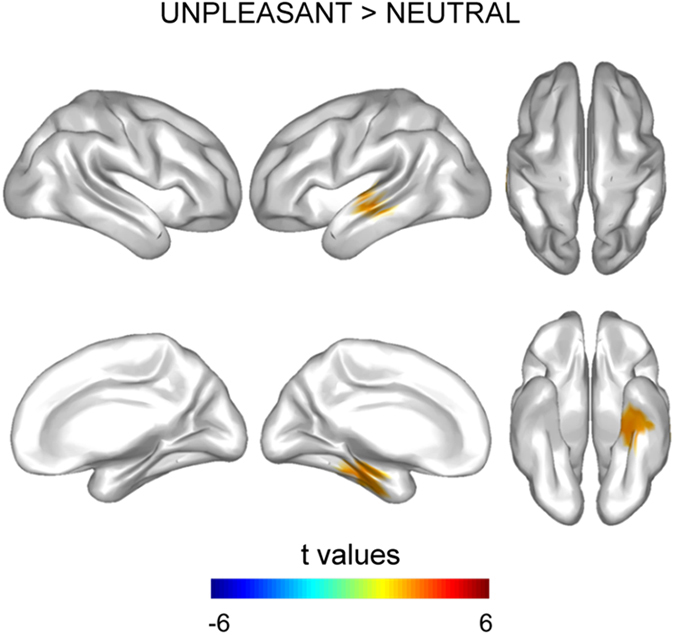
Cortical distribution of statistical differences in brain activity between 280–320 ms. Unpleasant distraction enhanced brain activity in the LTL, the MTL and the VTL.

Unpleasant distraction significantly enhanced activity in a left cortical cluster (*p* = 0.04) at 280–320 ms, relative to neutral distraction. This cluster of activity was comprised of regions over the superior (STC), lateral (LTC), medial (MTC) and ventral temporal cortex (VTC) (see Fig. 3 for cortical distribution of statistical differences in brain activity and Table S1 for specific cortical regions included in the cluster, as defined in the AAL atlas^39^).

### Cognitive control of emotional distraction at late latencies

**Figure 4:**
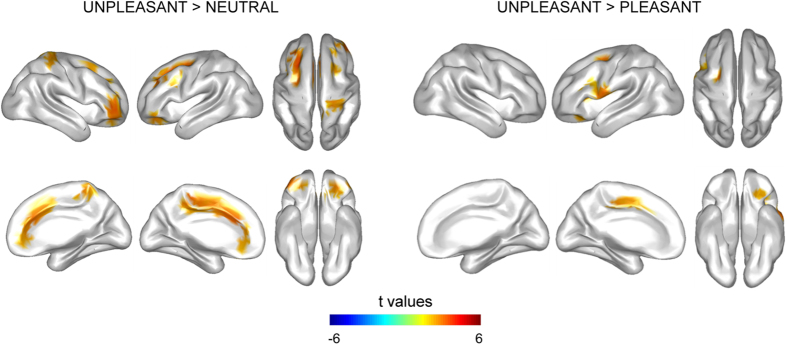
Cortical distribution of statistical differences in brain activity between 360–455 ms. Unpleasant distraction enhanced brain activity in the DLPFC, the VLPFC, the OFC, the MPFC, the PosFC and the PC when compared with both neutral and pleasant distraction.

Negatively valenced emotional distraction significantly enhanced brain activity at 360–455 ms relative to neutral and positively valenced distractors. When compared with neutral distractors, unpleasant distraction increased brain signal in a bilateral cluster (*p* = 0.006) distributed over the DLPFC, the VLPFC, the OFC, the MPFC and the PosFC, as well as over the PC and the medial parietal cortex (MPC). Unpleasant distractors also increased cortical activity when compared with pleasant ones, in a left lateralized cluster (*p* = 0.02) which included the DLPFC, the VLPFC, the OFC, the MPFC, the PosFC and PC (see Fig. 4 for cortical distribution of statistical differences in brain activity and Table S1 for specific cortical regions included in the cluster, as defined in the AAL atlas^39^).

### Brain activity and behavioral performance

**Figure 5:**
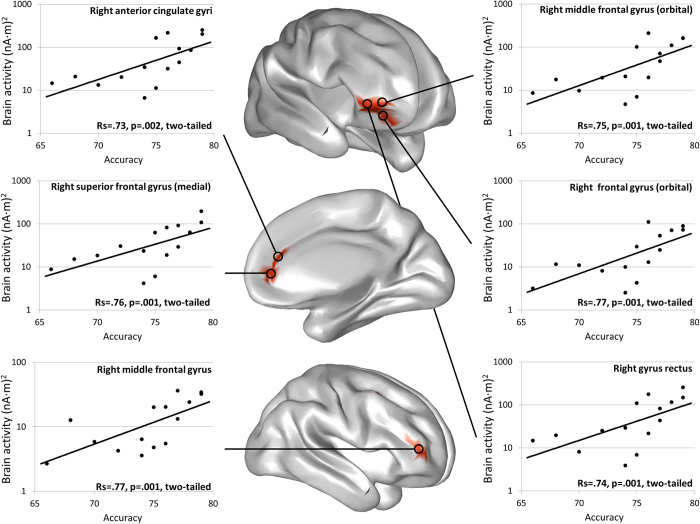
The role of the prefrontal cortex in coping with emotional distraction. The brain activity in specific regions of the DLPFC, the MPFC and the OFC at the 360–455 ms latency of unpleasant distraction processing positively correlated with successful performance at the recognition stage of that condition of the WM task.

No significant correlations for any condition were found during the first and second temporal windows [False discovery Rate (FDR) corrected *q* = .05, for all correlations]. In the third temporal window, activity in specific regions of the right OFC, DLPFC and MPFC in unpleasant distraction positively correlated with accuracy, so that volunteers with greater activity over those prefrontal cortices were those who performed better during the recognition stage of that condition (*p* < .05, FDR corrected *q* = .05, for all the reported correlations) (see Fig. 5 for specific localizations of brain regions, scatter plots, correlation coefficients and significance values). No significant correlations were found between brain activity and accuracy for neutral or for pleasant distraction (FDR corrected *q* = .05, for all correlations), during this time window.

## Discussion

Previous studies have shown that emotional stimuli can impair the retention of task-relevant information when they are presented as distractors in WM. Most of those studies have focused on the effect of unpleasant emotional distractors, and their power as interfering stimuli has been linked to its biological relevance for survival^14^,^15^,^16^,^17^. Results of the present study show that other type of emotional distractors are not so able to interfere WM maintenance, as pleasant distractors did not affect WM retention more than neutral ones. Yet, our volunteers rated pleasant pictures as less arousing than unpleasant stimuli, and therefore the arousal dimension might still have a potential contribution. Further studies should account for this issue, trying to keep the emotional distraction conditions equal in arousal, not only based on their normative values but also in the participant’s subjective ratings.

However, the main objective of the present work was to unravel the temporal profile of the brain mechanism that underlies the cognitive control of emotional distraction in WM. We identified three temporal windows of interest, in which differences of activity between distractor types arose. During the earliest significant temporal window, both types of emotional distraction increased the brain activity when compared with neutral distraction, specifically over frontal cortices including prefrontal regions such as the DLPFC, the VLPFC, the OFC, and the MPFC. Many studies have highlighted the central role of top-down modulation in visual processing^40^, since the prefrontal cortex has been reported to be active in visual recognition^41^,^42^,^43^ and, more interestingly, during visual processing of emotional stimuli^44^,^45^,^46^,^47^,^48^. Moreover, activity in the prefrontal cortex during both emotional and non-emotional visual stimulation has been shown at very early latencies, about 100 ms after the onset of the stimuli^41^,^44^,^49^,^50^. Such an early response of the prefrontal cortex during visual processing has been interpreted as a top-down facilitation mechanism in object recognition. This top-down processing of partial visual information reduces the possible interpretations of the input and minimizes the amount of time required for object recognition, which may be extremely helpful when the visual stimulus represents biologically relevant information. According to this model proposed by Bar^40^, increased prefrontal activation at early latencies of both pleasant and unpleasant distraction processing would reflect a top-down mechanism that may improve our preparation to adaptively respond to linked-to-survival stimuli. Particularly, enhanced activity in the OFC, which has been related to guessing processes and generation of expectations^51^,^52^,^53^,^54^, would be crucial for the rapid identification of biological information, as such contained in emotional distractors.

Our results also identified a later significant temporal window, in which unpleasant distraction increased the brain activity when compared with both pleasant and neutral distraction. Differences in activity were distributed over the DLPFC, the VLPFC, the OFC, the MPFC and the PosFC, as well as over the PC. Activity in the DLPFC, the ACC and the PC has been largely related to successful performance in WM tasks^20^,^21^,^22^,^23^,^24^,^25^, and these regions, along with the VLPFC and the PosFC, have been reported as important areas for interference resolution and inhibition of prepotent responses^23^,^55^,^56^,^57^,^58^,^59^. Further analysis of our data revealed that activity in specific regions of the right DLPFC, the right ACC and the right OFC -including a portion of cortex that overlaps the inferior section of the VLPFC- positively correlated with successful recognition after unpleasant distraction. Although the VLPFC and specific regions of the DLPFC and the MPFC have previously been linked to mechanisms of coping with unpleasant emotional distraction in WM^14^,^15^,^16^,^17^,^18^,^19^,^33^, the OFC has not been extensively related to successful control of such distraction^14^. However, it does play an important role in tasks that require inhibition of prepotent responses^60^,^61^,^62^, especially when such responses were established on their previous reward value^63^. Taking into account that the attentional capture by emotional distraction may be seen as a prepotent attentional response that should be overridden in our task, it is conceivable that the OFC appeared to be implicated in inhibition of such an attentional response. Altogether, these results are in consonance with previous fMRI studies that have highlighted the implication of the VLPFC in coping with unpleasant emotional distraction^14^,^16^,^17^,^19^,^33^, and extend the evidence of activity in the DLPFC, the MPFC and the OFC in relation to the cognitive control of unpleasant distractors in WM^14^,^15^,^16^,^18^. Furthermore, that significant enhancement of activity about 360–455 ms, when effective control of distraction seemed to take place, was restricted to unpleasant distraction. This fact suggests that such a control mechanism may be specially engaged during unpleasant distraction. The absence of differences between pleasant and neutral distractors also suggests that coping with positively valenced distractors would not require additional resources to those engaged when coping with neutral ones, as reflected by an equivalent WM performance at the behavioral level.

Finally, our results also revealed a third significant temporal window that arose between those temporal windows commented above. Pairwise comparisons revealed that unpleasant distraction enhanced the brain activity over superior, lateral, medial and ventral surfaces of the left temporal lobe, when compared with neutral distraction. Dolcos and cols^19^. have recently proposed that the impairing effect of unpleasant emotional distraction in WM may co-occur with the consistently observed effect of enhanced episodic memory for emotional events^7^,^8^. We proposed that the higher activity over the left temporal lobe in the unpleasant distraction condition of our task might be reflecting this effect of episodic memory enhancement for the unpleasant distractors themselves. However, this interpretation is only tentative, as we did not test the subsequent episodic memory for the distractors in our volunteers, and therefore we were not able to test a potential relation between temporal lobe activity and subsequent episodic memory for the distractors.

Although most of the previous studies in this field have identified a dissociable pattern of activity between dorsal cortical regions and ventral brain areas^14^,^15^,^16^,^17^,^18^,^19^ when coping with emotional distraction, our results did not show deactivations over the DLPFC and PC. Moreover, the dorsal activity in our study was always higher for emotional distractors than for neutral ones. However, all the previous studies that found such dorsal deactivations employed fMRI for their experiments. As the functional signal recorded in fMRI has a different origin than the MEG signal, since the first relies in the slow hemodynamic response while the latter records the very fast electromagnetic changes^64^,^65^, our results may not be straightforwardly compared with previous fMRI results. On top of that dissimilarity in the origin of the signal, our study also focused on a different stage of the cognitive mechanism of coping with emotional distraction. While previous fMRI findings reflected a late stage of such process (approximately between 6 to 10 seconds following the onset of the distractor), our findings reflect the first second following the onset of the distractor, an early stage that remains inaccessible to fMRI investigations due the slowness of the hemodynamic response. Therefore, results from the present study should be taken as a complement to the existent literature, rather than as a discrepancy, and also as an extension to the wealthy literature that links the DPFC with emotion processing^32^. In spite of these discrepancies, the enhanced activity over ventral prefrontal cortices for emotional distraction in our results is consistent with previous literature concluding that the right VLPFC is critically engaged in coping with emotional distraction^66^. Further, our results suggest that specific regions of the right OFC-VLPFC would also be important for overriding the emotional distraction.

The present study reveals for the first time the temporal dynamics of the brain mechanisms that underlie our capacity to deal with emotional distractors in WM. At the very early latencies of the distractor processing, prefrontal mechanisms are engaged for the rapid detection of both pleasant and unpleasant emotional distraction. Later in the processing, unpleasant distractors seem to recruit a specific cognitive control mechanism when compared with neutral and pleasant distractors. Such a mechanism depends on activity over the DLPFC, the MPFC and the OFC. Finally, in the time between the early detection and the effective control of the emotional distraction the increased activity in the temporal lobe, especially in the MTL, might be reflecting the well-known enhancement memory effect for emotional materials. The present findings contribute to our knowledge regarding the brain mechanisms of coping with emotional distraction in WM, and clarify for the first time the temporal dynamics of those cognitive control mechanisms.

## Methods

### Participants

Participants were 19 students from the Camilo José Cela University of Madrid. The project was approved by the institutional Review Committee of the Center for Biomedical Technology (Technical University of Madrid and Complutense University of Madrid) and the procedure was performed in accordance with approved guidelines and regulations. All participants gave written informed consent. Data from 4 volunteers was excluded from the analysis because of failure of the behavioral response recording system (3 participants) or for performance lower than 60% at any condition of the WM task and/or an insufficient amount of artefact-free trials for source reconstruction (1 participant). Hence, analyses of the behavioral and MEG data correspond to 15 volunteers (7 males and 8 females. Mean age 20.06 years and a range between 18 and 29 years). They had normal or corrected-to-normal vision. They all completed the Spanish version of the Spielberger State-Trait Anxiety Inventory for Adults^67^ (mean State score 15.07, sd 7.17; mean Trait score 12.36, sd 5.88) and the Beck Depression Inventory^68^ (mean score 6.46, sd 5.10). Participants received course credits for their time.

### Materials

Items at encoding and recognition stages consisted of colored images of neutral faces. An oval mask was applied along the contours of the faces to remove ears and hair and avoid any potential non-face specific cues. A pair of faces was presented at the encoding stage while just one face was displayed at the recognition stage. Faces were assigned to different experimental conditions across subjects. For the interfering items presented at the maintenance period, the International Affective Picture System (IAPS)^69^ was scanned to obtain three sets of images that formed the pleasant, neutral and unpleasant distractors. Pictures in the pleasant and unpleasant distraction conditions were selected as to differ in valence but not in arousal. 48 pictures between 8.5–6.5 valence and 7.5–5.5 arousal formed the pleasant condition. Another 48 pictures between 3.5–1.4 and 6.6–4.3 formed the unpleasant condition. Finally, 48 medium-valenced (5.5–4.0) and low-arousing (3.7–1.7) pictures were selected for the neutral distraction condition (see Table 1 for mean normative values).

### Procedure

**Figure 6:**
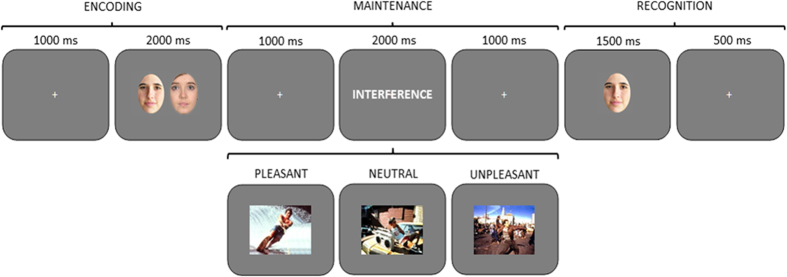
Diagram of the delayed-recognition WM paradigm. Three types of distractors (pleasant, neutral and unpleasant) were pseudorandomly presented during the maintenance stage. Volunteers were trained to learn and maintain the pair of faces into WM, look at the distracter, and then decide whether the face at the recognition stage is one of the two previously encoded or not, by pressing one of two keys. Photographs representing distractors in this Figure were also extracted from the International Affective Picture System (IAPS)^69^.

A delayed-recognition WM paradigm with three experimental conditions, pleasant, neutral and unpleasant interference was used (see Fig. 6). Each trial began with a 1000 ms intertrial interval (ITI), followed by the presentation of a pair of faces for 2000 ms (encoding phase). After a 1000 ms blank screen, an interfering stimulus was displayed for 2000 ms, followed by another 1000 ms blank screen (maintenance phase). Next, just one face appeared on the screen for 1500 ms, followed by a 500 ms blank screen (recognition stage). Participants had to decide whether the face at the recognition stage had been one of the two previously encoded or not, by pressing one of two buttons.

Each experimental condition included 96 trials in order to achieve an adequate signal-to-noise ratio for subsequent brain source estimation. Therefore, each one of the 48 previously selected interfering pictures was employed in two different trials. To avoid inducing long-lasting mood states, the order of trials was constrained so that no more than three trials of the same condition were consecutively presented. To prevent any potential habituation effect, the two presentations of the same interfering picture were separated by a minimum of thirty trials. Before the experiment, all the volunteers underwent four training trials in order to ensure that they completely understood the task. These trials were not used later in the analysis. Once the WM paradigm was completed, all the pictures used as interference were presented to the participants out of the MEG system, and they were asked to rate them regarding to emotional valence and arousal, using the Self-Assessment Manikin (SAM) self-report scale^70^. Participants were allowed to see each picture as long as they wanted, and the order of presentation of the pictures was also constrained in the same way, but in a different sequence, than for the WM task.

### Data acquisition and preprocessing

MEG data was continuously recorded (1000 Hz sample rate, 0.01–330 Hz online filter) during the performance of the WM task using a 306-channel (102 magnetometers and 204 planar gradiometers) system (Elekta©, VectorView), inside a magnetically shielded room (Vacuumschmelze GmbH, Hanau, Germany). Activity in electrooculogram channels was also recorded to keep track of ocular artefacts. Maxfilter software (version 2.2., Elekta Neuromag) was used to remove external noise with the temporal extension of the signal space separation method^71^.

Raw data was band-pass filtered with low and high cutoffs of 1 and 45Hz, respectively, and segmented for each trial beginning 300 ms prior to distractor onset and continuing for 2,000 ms. Baseline correction was performed for each trial, using the 300 ms prior to distractor onset. Epochs were discarded from the analysis when containing eye, muscular or movement artefacts identified by visual inspection, or amplitudes higher than 3 pT.

The output of this preprocessing stage was a set of artefact-free trials for each condition and for each MEG channel. Only those trials associated with successful WM performance were included in further analyses. For the subsequent analysis we decided to use exclusively the magnetometer data, since magnetometers enable the analysis of deeper sources such as the orbital part of the frontal lobe and the cingulate cortex, which have been reported active in previous studies involving memory control mechanisms and emotional processing^66^. The whole analysis was performed using the Fieldtrip toolbox (http://fieldtrip.fcdonders.nl/) in combination with in-house-MATLAB^©^-code (The Mathworks, Natick, MA).

### Statistical analysis at sensor level

A minimum of 52 artifact-free epochs were averaged to obtain an event related field (ERF) for each participant and condition. To determine the time windows and channel locations of significant differences in magnetic amplitude between the three distraction conditions, dependent samples F-tests were used. To control for the familywise error rate in the context of multiple comparisons (time points and sensors), a cluster-based nonparametric permutation statistic^72^ was performed. Accordingly, clusters of channels and time samples with significant differences (*p* < 0.05) were created by temporal and spatial adjacency (a cluster had to consist of minimum of two significant neighboring sensors). Then, a set of 2000 permutations was created by randomly assigning condition labels and F-values were computed for each permutation. A cluster was considered to have a significant effect if the sum of F-values in the original dataset was greater than the 95th percentile (*p* < 0.05) of the distribution of the corresponding values in the randomized data.

### Source reconstruction

Based on the statistical analysis of the ERF in sensor space, three time windows of interest showing significant results were established: 70–130 ms, 280–320 ms and 360–455 ms. To estimate the changes in brain activity that caused these differences, a source reconstruction in these time intervals was performed.

### Headmodels

A regular grid of 2471 points with 1cm spacing was created in the template Montreal Neurological Institute (MNI) brain^73^. An anatomical label was assigned to each grid point with the AAL atlas^39^, as implemented in the WFU software^74^. Then, this set of points was transformed into subject’s space and constituted the source locations. For that, an iterative closest point algorithm was used, that yielded a 4 × 4 matrix (translation, rotation and resizing) that transformed a standard MNI skin into the subject’s headshape. The forward model was solved with a local spheres method^75^.

### Beamforming

Source reconstruction was performed with Linearly Constrained Minimum Variance Beamformer^76^. We followed a common filter approach that would ease the comparison between conditions (pleasant, neutral and unpleasant distraction): the spatial filter’s coefficients were obtained from the average covariance matrix from trials belonging to all three conditions and then this filter was applied to each condition separately. This procedure is performed for each time window separately, so that the output of this source reconstruction step consists in a power estimate per source location, condition, time window and subject.

### Statistical analysis on source space

To identify which condition differs from each other in brain activity, dependent samples T-tests were performed. A clustering and permutation procedure was used to correct for multiple comparisons, using the sum of the T-values in the original dataset for the threshold, as introduced for the Statistical analysis at sensor level section. However, the clustering step groups now the spatially adjacent sources that show significant differences (*p* < 0.05), and employs their 3D coordinates for that grouping. 2000 permutations were used to obtain the final and corrected p-value.

### Correlation analysis between brain activity and behavioral performance

To further investigate the physiological meaning of the reported differences in brain activity while coping with emotional distraction, we segmented significant clusters into smaller regions as defined in the AAL atlas^39^ (see Table S1). Then, we identified the source that showed the maximal activity in each region and correlated that activity with task accuracy, for every experimental condition in each contrast and time window. In order to control for false positives in the context of multiple tests, we applied a False Discovery Rate control procedure^77^ with a *q* value of 0.05.

## Additional Information

**How to cite this article**: García-Pacios, J. *et al*. Early detection and late cognitive control of emotional distraction by the prefrontal cortex. *Sci. Rep.* **5**, 10046; doi: 10.1038/srep10046 (2015).

## Supplementary Material

Supporting Information

## Figures and Tables

**Table 1:** Mean normative values of pictures used in Second Study and mean subjective ratings of those pictures by our volunteers.

**Condition**	**IAPS Valence**	**IAPS Arousal**	**Subjective Valence**	**Subjective Arousal**
Pleasant	7.42 (0.33)	6.16 (0.49)	7.30 (1.00)	6.33 (0.94)
Neutral	4.93 (0.35)	2.71 (0.38)	5.14 (0.49)	3.61 (1.33)
Unpleasant	2.48 (0.52)	6.16 (0.41)	2.42 (1.03)	6.77 (0.93)

Standard deviations are shown in parenthesis.
