# Supplementary material for: Early detection and late cognitive control of emotional distraction by the prefrontal cortex
Source: Sci Rep. 2015 Jun 12;5:10046. doi: 10.1038/srep10046 (PMC4464367; doi:10.1038/srep10046)
Supplement: Supporting Information [file srep10046-s1.docx]

**EARLY DETECTION AND LATE COGNITIVE CONTROL OF EMOTIONAL DISTRACTION BY THE PREFRONTAL CORTEX**

**Supplementary Material**

**Javier García-Pacios ^a, b^, Pilar Garcés ^a^, David Del Río ^a, c^, Fernando Maestú ^a, c^**

**^a^** Laboratory of Cognitive and Computational Neuroscience. Center for Biomedical Technology (Technical University of Madrid and Complutense University of Madrid). Campus de Montegancedo. 28223, Pozuelo de Alarcón, Madrid, Spain

**^b^** Department of Psychology. Faculty of Health Sciences. Camilo José Cela University, Madrid. C/ Castillo de Alarcón, 49, Urb. Villafranca del Castillo. 28692, Madrid, Spain

**^c^** Department of Basic Psychology II. Complutense University of Madrid. Campus Somosaguas. 28223, Pozuelo de Alarcón, Madrid, Spain

**Corresponding author:** Javier García-Pacios

**Email**: javier.garciapacios@ctb.upm.es

**Telephone:** (+34) 913364642

Parcellation of significant clusters into smaller regions, as defined in the AAL atlas^39^. Segmentation was performed for clusters obtained from every statical contrast, within each temporal window of interest.

| **Left Hemisphere** | **Right Hemisphere** |
| --- | --- |
| ***70-130 ms Pleasant > Neutral*** |  |
| **DLPFC** | **DLPFC** |
| Superior frontal gyrus  Middle frontal gyrus  **VLPFC** | Superior frontal gyrus  Middle frontal gyrus  **VLPFC** |
| Inferior frontal gyrus (triangular part)  **OFC** | Inferior frontal gyrus (triangular part)  Inferior frontal gyrus (opercular part)  **OFC** |
| Superior frontal Gyrus (orbital part)  Middle frontal gyrus (orbital part)  Inferior frontal gyrus (orbital part)  Superior frontal gyrus (medial orbital part)  Gyrus rectus  **MPFC** | Superior frontal Gyrus (orbital part)  Middle frontal gyrus (orbital part)  Inferior frontal gyrus (orbital part)  Superior frontal gyrus (medial orbital part)  **MPFC** |
| Superior frontal gyrus (medial part)  Anterior cingulate gyri  Median cingulate gyri  **PosFC** | Superior frontal gyrus (medial part)  Anterior cingulate gyri  Median cingulate gyri  **PosFC** |
| Precentral gyrus  Supplementary motor area | Precentral gyrus  Supplementary motor area |
| ***70-130 ms Unpleasant > Neutral*** | |
| **DLPFC** | **MPFC** |
| Superior frontal gyrus  Middle frontal gyrus  **VLPFC** | Anterior cingulate gyri  **OC** |
| Inferior frontal gyrus (triangular part)  **OFC** | Inferior occipital gyrus  Middle occipital gyrus |
| Superior frontal Gyrus (orbital part)  Middle frontal gyrus (orbital part)  Inferior frontal gyrus (orbital part)  Superior frontal gyrus (medial orbital part)  Gyrus rectus  **MPFC** |  |
| Superior frontal gyrus (medial part)  Anterior cingulate gyri  Median cingulate gyri  **PosFC** |  |
| Precentral gyrus  Supplementary motor area |  |
|  |  |
| ***280-320 ms Unpleasant > Neutral*** |  |
| **STL**  Superior temporal gyrus  Heschl gyrus  **LTC** |  |
| Middle temporal gyrus  Inferior temporal gyrus  **MTL** |  |
| Hippocampus  Parahippocampal gyrus  **VTL** |  |
| Fusiform gyrus |  |
|  | |
| ***360-455ms Unpleasant > Neutral*** | |
| **DLPFC** | **DLPFC** |
| Superior frontal gyrus  Middle frontal gyrus  **VLPFC** | Superior frontal gyrus  Middle frontal gyrus  **VLPFC** |
| Inferior frontal gyrus (triangular part)  Insula  **OFC** | Inferior frontal gyrus (triangular part)  **OFC** |
| Superior frontal gyrus (orbital part)  Middle frontal gyrus (orbital part)  Inferior frontal gyrus (orbital part)  Superior frontal gyrus (medial orbital part)  Gyrus rectus  **MPFC** | Middle frontal gyrus (orbital part)  Inferior frontal gyrus (orbital part)  Superior frontal gyrus (medial orbital part)  **MPFC** |
| Superior frontal gyrus (medial part)  Anterior cingulate gyri  Median cingulate gyri  **PosFC** | Superior frontal gyrus (medial part)  Anterior cingulate gyri  Median cingulate gyri  **PosFC** |
| Precentral gyrus  Paracentral lobule  Supplementary motor area | Precentral gyrus  Paracentral lobule  Supplementary motor area |
| **PC**  Postcentral gyrus  Precuneus | **PC**  Postcentral gyrus  Precuneus  Inferior parietal gyrus |
|  | |
| ***360-455ms Unpleasant > Pleasant*** | |
| **DLPFC** |  |
| Superior frontal gyrus  Middle frontal gyrus  **VLPFC** |  |
| Inferior frontal gyrus (triangular part)  Inferior frontal gyrus (opercular part)  Insula  **OFC** |  |
| Middle frontal gyrus (orbital part)  Inferior frontal gyrus (orbital part)  **MPFC** |  |
| Anterior cingulate gyri  Median cingulate gyri  **PosFC** |  |
| Precentral gyrus  Paracentral lobule  Supplementary motor area  Rolandic operculum |  |
| **PC**  Postcentral gyrus |  |
